# Factors associated with mortality in posterior reversible encephalopathy syndrome: a nationwide analysis

**DOI:** 10.1007/s10072-026-08916-6

**Published:** 2026-02-24

**Authors:** Ali Al-Salahat, Richard Cheung, Danielle B. Dilsaver, Amelia Pham, Ripudaman Kahlon, Muhammad Roshan Asghar, Rohan Sharma

**Affiliations:** 1 Neurology Department, Creighton University, Nebraska, USA; 2 School of Medicine, Creighton University, Nebraska, USA; 3 Department of Clinical Research and Public Health, Creighton University, Nebraska, USA

**Keywords:** Posterior leukoencephalopathy syndrome, Posterior reversible encephalopathy syndrome, Hypertensive encephalopathy, Hypertensive crisis, Hospital mortality, Mortality, Healthcare disparities

## Abstract

**Background:**

To identify demographic and clinical factors associated with in-hospital mortality among hospitalizations of adults with posterior reversible encephalopathy syndrome (PRES) using the National Inpatient Sample (NIS) in the United States (US).

**Methods:**

Hospitalizations for PRES in patients ≥ 18 years were abstracted from the NIS (2016–2022). To identify clinical factors associated with in-hospital mortality, univariable logistic regression models were estimated and significant predictors were retained in a multivariable logistic regression model. Collinearity was assessed via variance inflation factors. Association was quantified using adjusted odds ratios (aOR).

**Findings:**

There were an estimated 75,830 PRES hospitalizations, of which, 3,665 in-hospital deaths occurred (4.8%). Decedents were older, had longer stays, and higher costs. In the adjusted model, the strongest factors associated with increased mortality were respiratory failure (aOR 5.43; 95% CI 4.40–6.71), sepsis (aOR 2.45; 95% CI 2.02–2.96), and ischemic stroke (aOR 2.25; 95% CI 1.86–2.72). Additional independent risk factors included cerebral edema, coma, intracerebral hemorrhage, severe liver disease, subarachnoid hemorrhage, kidney disorders, status epilepticus, encephalitis/encephalomyelitis, malignancy, complications of transplanted organs, and COVID-19. Documented hypertensive crisis (OR 0.63; 95% CI: 0.52 to 0.75) and history of epilepsy/seizures (OR 0.67; 95% CI: 0.55 to 0.81) were associated with lower odds of death.

**Conclusion:**

Respiratory failure, sepsis, and cerebrovascular complications drive in-hospital mortality in PRES. Early airway protection, aggressive supportive care, and prompt neurovascular evaluation for high-risk patients may improve outcomes. Prospective studies with granular clinical and imaging data are needed to refine prognostic models.

**Supplementary Information:**

The online version contains supplementary material available at 10.1007/s10072-026-08916-6.

## Introduction

Posterior reversible encephalopathy syndrome (PRES) is a neurovascular condition characterized by acute or subacute onset of a variable constellation of symptoms including but not limited to headache, seizures, encephalopathy and visual disturbance [1, 2]. This syndrome was initially codified as a distinct entity by Hinchey et al. almost three decades ago [2]. It is believed to occur secondary to impairment of autoregulation, most commonly in the setting of severely elevated blood pressure [1–4]. Even though it is considered a reversible condition with a good prognosis if treated promptly, studies have reported non-trivial rates of morbidity and mortality [1, 5]. Analysis of the Berlin PRES study reported a mortality rate reaching 11.2% associated with PRES [5]. Factors associated with mortality in patients with PRES include altered level of consciousness, seizures, sepsis, acute kidney injury, and intracerebral hemorrhage [5, 6]. However, previous studies examining factors associated with PRES-related mortality were limited.

A clearer, population-level understanding of the factors associated with in-hospital mortality in PRES is needed to improve risk stratification, guide monitoring and treatment and identify health-system or demographic disparities that may influence outcomes. Using a large, nationally representative inpatient database, we examined demographic, neurological and medical co-morbidities associated with in-hospital mortality among hospitalizations with PRES. Our primary aim was to identify factors associated with mortality that can inform bedside prognostication and generate hypotheses for prospective, patient-level research.

## Methods

### Data source

This study followed the RECORD reporting guidelines. Data were extracted from the National Inpatient Sample (NIS) from 2016 to 2022. The NIS is a large, publicly available, all-payer database with inpatient discharge records from hospitals throughout the US [7]. More specifically, the data were de-identified administrative claims data that ensured patient anonymity and confidentiality. As such, the study was deemed Non-Human Subject Research by the Institutional Review Board (InfoEd Record Number: 2005564).

### Cohort identification

The study population consisted of hospitalizations of patients carrying a diagnosis of PRES based on the *International Classification of Disease*,* Clinical Modification*,* Tenth Revision* code (ICD-10-CM: I67.83). Hospitalizations of patients less than 18 years of age were excluded as well as hospitalizations with a primary reason for hospitalization that was unrelated to a PRES diagnosis. Hospitalizations for the following unrelated *Medicare Severity Diagnosis Related Groups (MS-DRGs)* were excluded: 113–125 (eye disorders), 135–159 (Ear, nose and throat), 453–566 (musculoskeletal), 570–607 (skin), 707–730 (male reproductive disorders), 789–798 (newborn related), 876–887 (mental conditions).

### Variables of interest

The outcome variable was in-hospital mortality. Descriptive characteristics included age, biological sex (male, female), race/ethnicity (White, Black, Hispanic, other), primary payer (Medicare, Medicaid, Private, other), median household income quartile (1st -25th, 26th -50th, 51st -75th, 76th -100th ), weekend admission, hospitalization cost, hospitalization length of stay, hospital location and teaching status (rural, urban teaching, urban non-teaching), and hospital bed size (small, medium, large). Among the neurological comorbidities of interest were epilepsy and seizures (EPS), status epilepticus (SE), ischemic stroke (IS), non-traumatic subarachnoid hemorrhage (SAH), intracerebral hemorrhage (ICH), cerebral edema (CE), encephalitis, myelitis, or encephalomyelitis (EM) that is inflammatory or infectious, coma (upon arrival to emergency room, upon inpatient admission, 24 or more hours after admission). Other medical conditions of interest included myocardial infarction, congestive heart failure, peripheral vascular disease, stroke, dementia, chronic obstructive pulmonary disease, rheumatic disease, peptic ulcers, liver disease, diabetes, renal disease, plegia/paralysis, malignancy, metastatic tumor, acute renal failure, kidney disorders, sepsis, hypertensive crisis, reversible cerebral vasoconstriction syndrome, hypertensive encephalopathy, respiratory failure, coronavirus disease 2019 (COVID-19), toxic encephalopathy, primary hypertension, hypertensive heart and chronic kidney disease, aspiration pneumonitis, urinary tract infection, alcohol related disorders, connective tissue diseases with systemic involvement, systemic lupus erythematosus, hematological disorders, acute pancreatitis, body mass index of 30 or above, transplanted organ and tissue, complications of transplanted organs and tissue. Supplemental Table I provides a complete list of all the neurological and medical comorbidities or associated conditions that were screened in this study.

### Statistical analysis

Descriptive characteristics were stratified by mortality status and compared via Rao-Scott chi-square test or t-tests, depending on whether the variables were categorical or continuous. The primary aim was to identify clinical and demographic disparities associated with PRES-related in-hospital mortality (PRES-RM). To evaluate associations between neurological and medical comorbidities and PRES-RM, we estimated logistic regression models. First, simple logistic regression models were estimated to assess whether the odds of PRES-RM varied by neurological and medical comorbidities; comorbidities were specified “a priori”. A complete list of comorbidities is provided in Supplemental Table II. Next, the statistically significant comorbidities (*p* < 0.05) were included in a multivariable logistic regression model, and redundant variables were removed based off their variance inflation factor (VIF) and degree of freedom (DF). Comorbidities were then sorted by t-value as a proxy for statistical importance. All analyses were conducted in SAS v. 9.4. Two-tailed *p* < 0.05 was considered statistically significant.

## Results

### Baseline characteristics and demographics

**Fig. 1:**
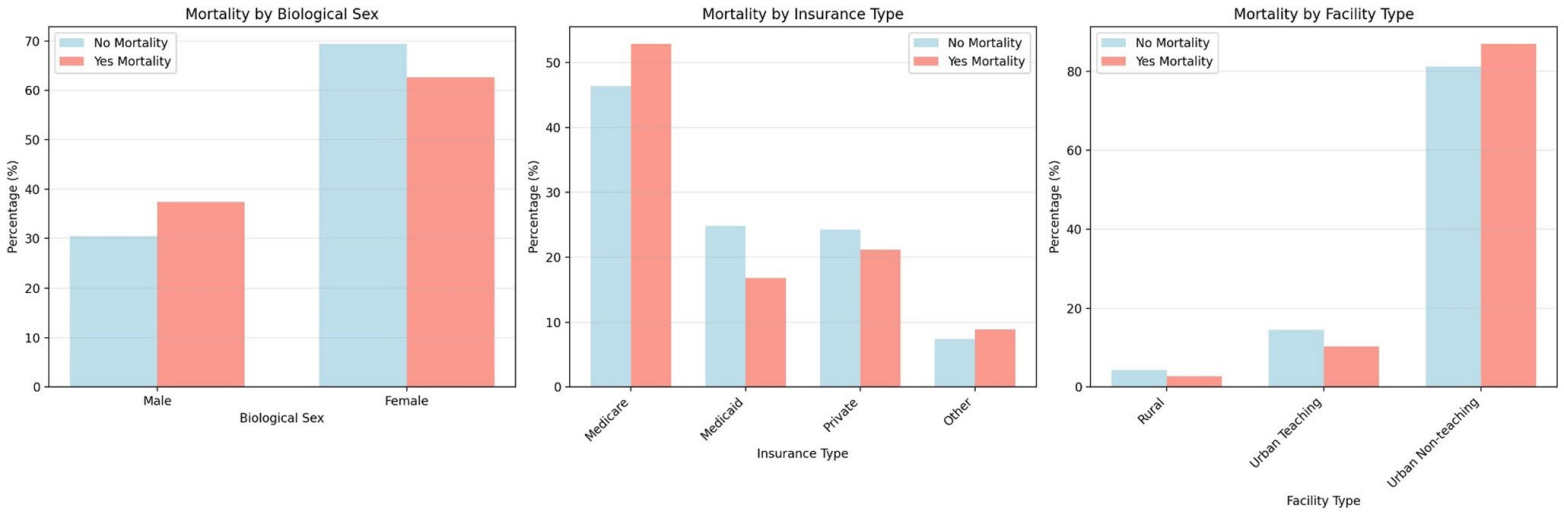
Demographic disparities in sex, insurance type and facility type based on mortality

From 2016 to 2022, there were an estimated 75,830 (95% CI: 74,336 to 77,324, Unweighted N: 15,166) hospitalizations with PRES. Only 403 hospitalizations were excluded as they carried DRG codes unrelated to PRES. Of PRES hospitalizations meeting inclusion criteria, there were an estimated 3,665 deaths (4.83%, 95% CI: 4.49% to 5.18%) with 62.62% and 37.38% involving female and male patients, respectively. Table 1 provides baseline descriptive characteristics stratified by mortality status. Notably, female sex, insurance type, hospital teaching status and hospital size varied by morality status (Fig. 1). More specifically, the PRES-RM cohort was more frequently male, on Medicaid, and at urban-teaching facilities (Table 1; Fig. 1).

### Clinical variables and selection

**Fig. 2:**
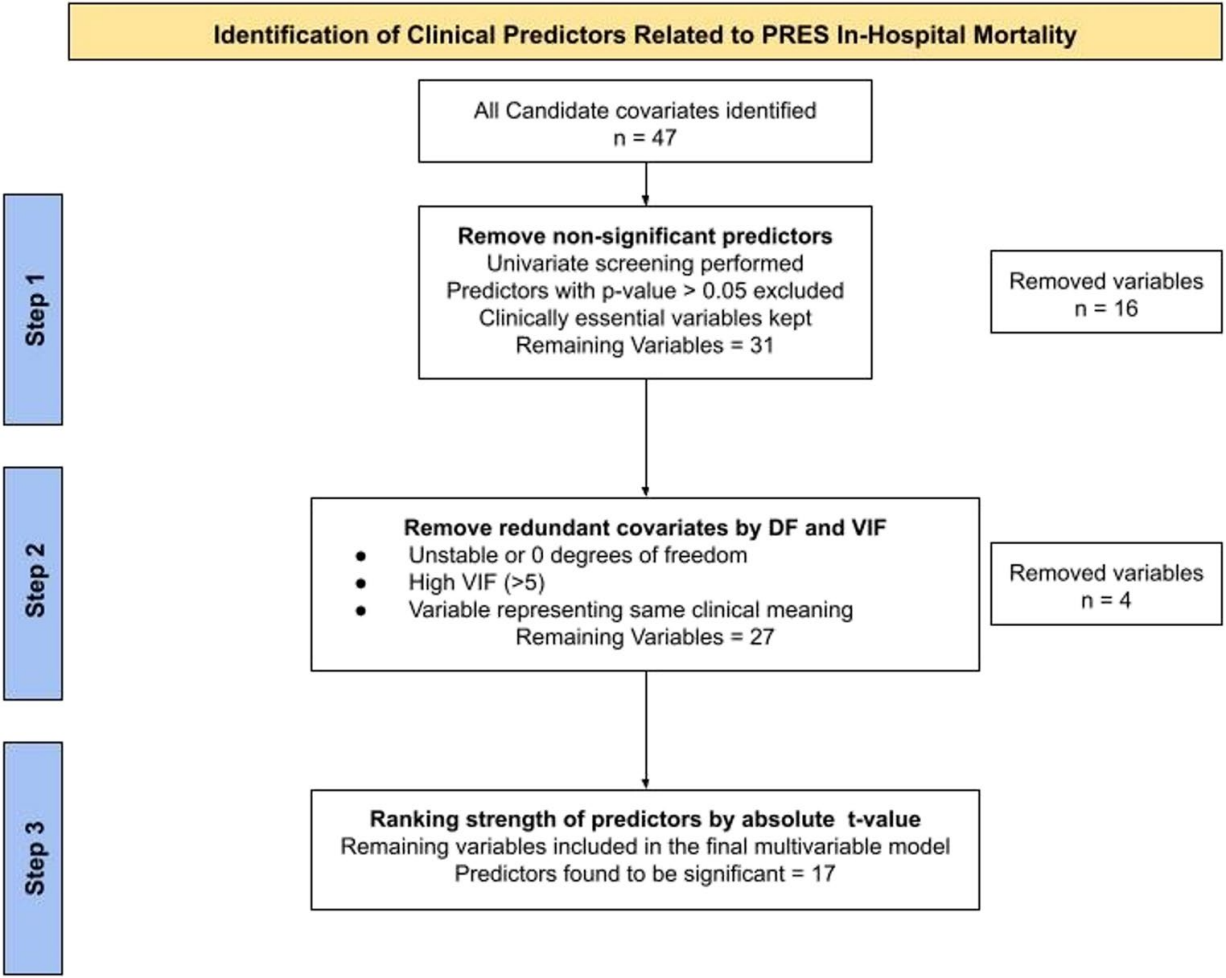
Flow chart detailing the stepwise approach to identifying and selecting significant clinical predictors associated with PRES mortality. DF = degrees of freedom, n = count, VIF = variance inflation factor

**Fig. 3:**
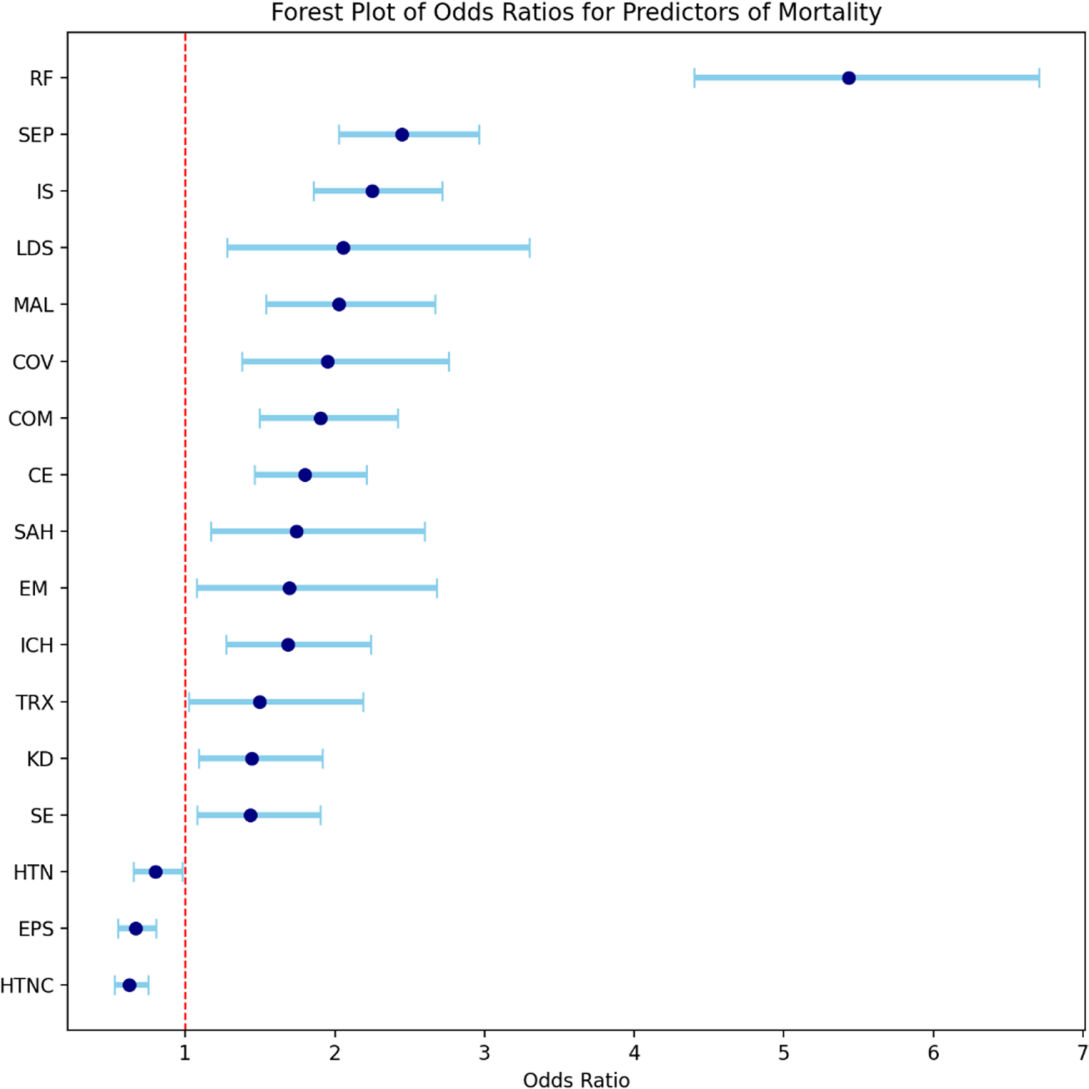
Forest plot showing the odds ratios of the significant clinical predictors in PRES-related mortality (error bar represents 95% confidence intervals). HTC = hypertensive crisis, EPS = History of Epilepsy and Seizures, HTN = history of essential hypertension, SE = Status epilepticus, KD = Kidney disorders, TRX = complications in transplanted organs, ICH = intracerebral hemorrhage, EM = encephalitis/encephalomyelitis, SAH = subarachnoid hemorrhage, CE = Cerebral edema, COM = coma, COV = covid-19, MAL = malignancy, LDS = liver disease, IS =ischemic stroke, SEP = sepsis, RF = respiratory failure

The process of identifying predictors started with 47 conditions. Supplemental Table II provides the frequency of all 47 conditions stratified by mortality status. Supplemental Table III provides the unadjusted odds ratios for all 47 conditions. Of the 47 conditions, 16 non-significant predictors were removed from the multivariable model along with conditions that had less than 10 observed deaths (due to the NIS Data Use Agreement). Based on VIF and degrees of freedom, 4 variables were removed due to redundancy resulting in a total of 27 clinical variables in the multivariable logistic regression model. Figure 2 provides a flow chart illustrating the identification and selection of clinical conditions. After estimating the multivariable logistic regression model clinical predictors were sorted by their absolute t-values (i.e., strength of association) as shown in Table 2. Figure 3 presents a forest plot of the 17 clinical predictors that were statistically associated with PRES-RM in the multivariable model (*p* < 0.05).

## Discussion

### Clinical factors associated with mortality

This study revealed several novel and important findings. First, respiratory failure had a strong association with PRES-RM, underscoring the central role of airway and ventilatory complications in the trajectory of patients with PRES. Respiratory compromise in this population reflects prolonged seizures, depressed consciousness, aspiration or multisystem critical illness. This is also reinforced by our finding that depressed level of consciousness was independently associated with elevated PRES-RM. Hence, the severity of cerebral dysfunction upon initial presentation has great prognostic value, highlighting the importance of early neurologic assessment and considering early intensive care for those presenting with decreased level of consciousness. The strong association between respiratory failure and level of consciousness and mortality highlights the need for early recognition and aggressive supportive care, such as timely airway protection and ventilatory support when indicated. Second, concomitant acute cerebrovascular events, such as ischemic stroke, ICH and SAH, were also independently associated with higher odds of PRES-RM. This finding is consistent with the fact that PRES can co-exist with or induce structural brain injury, and that these events substantially worsen prognosis. Clinicians should maintain a low threshold for neuroimaging when focal deficits or clinical deterioration occur.

An unexpected finding was that previous history of hypertension and the presence of documented hypertensive crises were associated with lower PRES-RM. A plausible explanation is that patients with known hypertension or hypertensive presentations may receive more rapid blood pressure recognition and targeted management, or that chronic hypertensive patients have adaptive cerebrovascular changes that alter the clinical course. Another plausible explanation is that normotensive PRES usually occurs in the setting of toxic medications, sepsis, renal failure, and circulating cytokines; these factors are associated with worse outcomes rendering the group without history of hypertension or hypertensive crisis on the less favorable side of comparison [8–11]. It is also noteworthy that previous studies did not directly analyze the association between prior history of hypertension and worse outcomes in PRES, or normotensive PRES versus hypertension-associated PRES [9–11]. Similarly, a history of epilepsy or prior seizures appeared protective, which may reflect pre-existing anti-seizure therapy and more rapid seizure control, thereby reducing the risk of status epilepticus and its complications.

Previous studies examining mortality from PRES found rates between 2 and 6% [12–14]. However, studies analyzing patients with PRES admitted to the intensive care unit found a mortality rate reaching 16% [12]. The Berlin PRES study found a higher in-hospital death rate of 11.2% among 151 patients [5]. Studies involving a Thai-Asian PRES population and another one with a German population found unfavorable outcomes in 22.8% and 42% of patients [6–15]. Our study found a mortality rate of 4.82%, which falls within the lower range compared to those studies. Several studies also attempted to identify factors that predict worse PRES outcomes. Impaired level of consciousness at presentation was a consistent independent predictor of poor outcomes and death in PRES [5, 6]. Extensive cerebral edema, as in our study, is also a strong predictor of death in PRES [1, 16]. Indeed, malignant PRES, defined by coma and cerebral edema, portends a poor prognosis [8]. Additionally, acute cerebrovascular events complicating PRES indicated poor prognosis in most previous studies and this was consistent with our findings [12–17].

### Demographic disparities in mortality

In our study, decedents were older, more often covered by Medicare, and more frequently admitted to large and urban non-teaching hospitals. Even though female sex was over-represented in our cohort, the proportion of males was higher among decedents. Median household income did not show statistically significant differences in mortality. Patients with Medicare are typically older with more comorbidities, hence vulnerable to complications, such as respiratory failure and sepsis. The increased mortality in urban non-teaching facilities could be due to lack of specialized neurocritical care team compared to teaching centers. This finding highlights the importance of strengthening transfer agreements and standardized critical-care pathways, that may eventually help reduce outcome variation among facilities. Previous studies did not find statistically significant differences in outcomes of PRES based on biological sex [18, 19]. There is evidence to suggest better outcomes in PRES related to pregnancy which could partially account for our findings [1, 20, 21]. The demographic data included in our study may assist clinicians and healthcare administrators to evaluate systems of care and target the vulnerable populations relating to PRES and ultimately reduce its mortality.

### Strengths and limitations

Strengths of this study include the large, nationally representative sample and the ability to examine a broad set of comorbidities and hospital characteristics. Several limitations temper interpretation. The analysis relied on ICD administrative codes that can be subject to misclassification bias. Also, the database lacks granular clinical detail, such as imaging findings, blood pressure trajectories, seizure burden, timing of interventions, and medications administration. The cross-sectional nature of inpatient discharge data precludes assessment of long-term outcomes and causal inference. Finally, residual confounding by unmeasured severity indicators is possible, such as those related to clinical examination, radiological findings and laboratory results.

## Conclusions

This population-level study identified respiratory failure, acute cerebrovascular complications, and depressed consciousness as key predictors of in-hospital mortality among patients with PRES, while suggesting that prior hypertension and epilepsy may be associated with lower in-hospital risk. These results support early aggressive supportive care and prompt neurovascular evaluation for high-risk patients, and they highlight the need for prospective studies that integrate clinical, radiographic, and treatment-timing data to refine prognostic models and guide interventions.

**Table 1:** Baseline and demographic characteristics of PRES hospitalizations stratified by mortality

Descriptives	In-hospital mortality		*p*-value
	No	Yes	
Hospitalizations, Weighted N (%)	72,165 (95.17)	3,665 (4.82)	
Age, mean ± SE (years)	55.59 ± 0.15	61.22 ± 0.54	< 0.001
Sex, Weighted N (%)			
Male	22,020 (30.51)	1,370 (37.38)	< 0.001
Female	50,120 (69.45)	2,295 (62.62)	
Missing	*	0 (0)	
Race/ethnicity, Weighted N (%)			
White	45,870 (65.55)	2,295 (66.33)	0.219
Black	15,085 (21.56)	650 (18.79)	
Hispanic	5,745 (8.21)	330 (9.54)	
Other	3,280 (4.69)	185 (5.35)	
Primary payer, Weighted N (%)			
Medicare	33,470 (46.38)	1,940 (52.93)	0.001
Medicaid	15,720 (24.78)	615 (16.78)	
Private	17,495 (24.24)	775 (21.15)	
Other	5,325 (7.38)	325 (8.87)	
Missing	155 (0.21)	*	
Median household income, Weighted N (%)			
0-25th percentile	23,895 (33.11)	1,215 (33.15)	0.787
26th -50th percentile	19,170 (26.56)	990 (27.01)	
51st -75th percentile	16,550 (22.93)	775 (21.15)	
76th -100th percentile	11,485 (15.91)	625 (17.05)	
Missing	1,065 (1.48)	60 (1.64)	
Weekend admission, Weighted N (%)	18,100 (25.08)	1,005 (27.42)	0.158
Hospitalization cost, mean ± SE (US dollars)	34,666 ± 526.02	84,358 ± 4,062.82	< 0.001
Length of stay, mean ± SE (days)	10.08 ± 0.11	16.61 ± 0.70	< 0.001
Hospital location-teaching status, Weighted N (%)			
Rural	3,070 (4.25)	100 (2.73)	< 0.001
Urban Teaching	10,465 (14.50)	375 (10.23)	
Urban non-teaching	58,630 (81.24)	3,190 (87.04)	
Hospital bed size, Weighted N (%)			
Small	10,590 (14.67)	420 (11.46)	0.002
Medium	18,145 (25.14)	800 (21.83)	
Large	53,430 (60.18)	2,445 (66.71)	

a. An * Asterisk denotes hospitalizations less than 10 in count and thus cannot be reported as per the National Inpatient Sample guidelines

b. N = weighted counts

c. SE = standard error

**Table 2:** Clinical variables sorted by their strength of association (i.e., absolute t-value)

Parameter	t-value	abs (t-value)	LCL	UCL	Pr > |t|
Respiratory failure	15.78	15.78	1.48	1.90	< 0.0001
Sepsis	9.20	9.20	0.70	1.09	< 0.0001
Ischemic stroke	8.32	8.32	0.62	1.00	< 0.0001
Cerebral edema (non-traumatic)	5.58	5.58	0.38	0.79	< 0.0001
Coma	5.27	5.27	0.40	0.88	< 0.0001
Hypertensive crisis	-5.08	5.08	-0.65	-0.29	< 0.0001
Malignancy	5.00	5.00	0.43	0.98	< 0.0001
Epilepsy and seizures	-4.19	4.19	-0.60	-0.22	< 0.0001
COVID-19	3.75	3.75	0.32	1.02	0.0002
Intracerebral hemorrhage (non-traumatic)	3.62	3.62	0.24	0.81	0.0003
Liver disease, severe	2.97	2.97	0.25	1.19	0.0029
Non-traumatic SAH	2.73	2.73	0.16	0.95	0.0063
Kidney Disorders	2.56	2.56	0.09	0.65	0.0104
Status epilepticus	2.48	2.48	0.08	0.64	0.0131
Encephalitis/myelitis/encephalomyelitis, inflammatory/infectious	2.26	2.26	0.07	0.99	0.0236
Primary hypertension	-2.14	2.14	-0.43	-0.02	0.0325
Complications of transplanted organs and tissue	2.08	2.08	0.02	0.78	0.0373

a. abs = absolute

b. t-value = t-statistic

c. aOR = adjusted odds ratio

d. CL = confidence limit

## Supplementary Information

Below is the link to the electronic supplementary material.

Supplementary Material 1

## Declarations

We confirm that authorship requirements have been met and the final manuscript was approved by all authors. We confirm that this manuscript has not been published elsewhere and is not under consideration by another journal. We confirm adherence to ethical guidelines and ethical approval (IRB) provided in the methods section in the manuscript. We followed the RECORDS reporting guidelines throughout the study.
