# Supplementary material for: Factors associated with mortality in posterior reversible encephalopathy syndrome: a nationwide analysis
Source: Neurol Sci. 2026 Feb 24;47(3):286. doi: 10.1007/s10072-026-08916-6 (PMC12929328; doi:10.1007/s10072-026-08916-6)
Supplement: Supplementary file 1 — Supplementary Material 1 [file 10072_2026_8916_MOESM1_ESM.docx]

**Supplemental Table I** Diagnosis codes used to identify conditions that were screened as potential predictors of PRES mortality

| **Conditions** | **ICD-10-CM/ICD-10-PCS** |
| --- | --- |
| Epilepsy and seizures | G40, R56.9 |
| Status epilepticus | G40.901, G40.911, G40.801, G40.803, G40.501, G40.401, G40.301, G40.311, G40.211, G40.201, G40.111, G40.101, G40.011, G40.001 |
| Ischemic stroke | I65.1, I63.22, I65.29, I63.139, I63.239, I65.09, I63.019, I63.119, I63.219, I63.20, I65.9, I63.59, I65.8, I63.50, I66.9, I63.40, I66.09, I66.19, I66.29, I63.30, I63.032, I63.033, I63.039, I63.031, I63.131, I63.132, I63.133, I63.231, I63.232, I63.233, I63.311, I63.312, I63.313, I63.319, I63.321, I63.322, I63.323, I63.329, I63.331, I63.332, I63.333, I63.339, I63.341, I63.342, I63.343, I63.349, I63.411, I63.412, I63.413, I63.419, I63.421, I63.422, I63.423, I63.429, I63.431, I63.432, I63.433, I63.439, I63.441, I63.442, I63.443, I63.449, I63.511, I63.512, I63.513, I63.519, I63.521, I63.522, I63.523, I63.529, I63.531, I63.532, I63.533, I63.539, I63.541, I63.542, I63.543, I63.549, I63.9, I65.21, I65.22, I65.23, I66.11, I66.12, I66.13, I66.21, I66.22, I66.23, I66.3, I66.8, I66.9, I63.011, I63.012, I63.013, I63.02, I63.12, I63.111, I63.112, I63.113, I63.211, I63.212, I63.213, I65.01, I65.02, I65.03, I63.00, I63.09, I63.19, I63.10, I63.29, I63.39, I63.49, I63.89, I63.8, I63.81 |
| Non-traumatic subarachnoid hemorrhage | I60.00, I60.01, I60.02, I60.10, I60.11, I60.12, I60.2, I60.30, I60.31, I60.32, I60.4, I60.50, I60.51, I60.52, I60.6, I60.7, I60.8, I60.9 |
| Intracerebral hemorrhage (non-traumatic) | I61.0, I61.1, I61.2, I61.3, I61.4, I61.5, I61.6, I61.8, I61.9 |
| Cerebral edema (non-traumatic) | G93.6 |
| Muscular/myotonic dystrophies | G71.1, G71.0 |
| Guillain-Barre syndrome | G61.0 |
| Unspecified cerebrovascular diseases | I67.89, I67.2, I67.81, I67.82, I67.89, I67.9, G45.9, I67.848, G45.1, G45.8, G45.0, G45.8, G46.1, G46.0, G45.2, G45.1, G45.8, G45.0, G46.2, G45.2, I67.81, I67.82, I67.89, I67.89, I68.0, I67.9, G46.5, G46.6, G46.7, G46.3, G46.4, G46.8, G45.8, G45.9, G45.3, H34.00 |
| Occlusion/stenosis of precerebral/cerebral arteries without infarction | I65, I66 |
| Meningitis, infectious | G00, G01, G02 |
| Encephalitis/myelitis/encephalomyelitis, inflammatory/infectious | G04, G05 |
| Intracranial/intraspinal abscess/granulomas/phlebitis/thrombophlebitis | G06, G07, G08 |
| Neurodegenerative conditions | G12.2, G20-G26, G30, |
| Neuroleptic malignant syndrome | G21.0 |
| Demyelinating conditions | G35-G37 |
| Peripheral neuropathies | G60-G65 |
| Migraine | G43 |
| Cerebral palsy | G80 |
| Autonomic disorders | G90 |
| Coma | R40.2 |
| Coma upon arrival to emergency room | R40.2112 |
| Coma upon admission | R40.2113 |
| Coma 24 hours or more after admission | R40.2114 |
| Myasthenia gravis, other neuromuscular junction disorders | G70 |
| Acute renal failure | N17 |
| Kidney disorders | N00-N19 |
| Sepsis | A40, A41, R65.2 |
| Systemic inflammatory response syndrome non-infectious | R65.1 |
| hypertensive crisis | I16 |
| Reversible cerebral vasoconstriction syndrome | I67.841 |
| Respiratory failure | J96 |
| COVID-19 | U07.1 |
| Toxic encephalopathy | G92 |
| Acute myocardial infarction | I21 |
| Primary hypertension | I10 |
| Resistant hypertension | I1A.0 |
| Secondary hypertension | I15 |
| Hypertensive heart and/or chronic kidney disease | I11, I12, I13 |
| Aspiration pneumonitis | J69 |
| Urinary tract infection | N39.0 |
| Alcohol related disorders | F10 |
| Connective tissue diseases with systemic involvement | M30-M36 |
| Systemic lupus erythematosus | M32 |
| Malignant neoplasms, including secondary | C76, C77, C78, C79, C80 |
| Hematological disorders | D50-D89 |
| Acute pancreatitis | K85 |
| Body mass index 30 or above | Z68.3, Z68.4 |
| Transplanted organ and tissue status | Z94 |
| Complications of transplanted organs and tissue | T86 |
| History of immune checkpoint medications | Z92.26 |
| Obstetric-related conditions and complications | O00-O99, O9A |
| **Procedure** | **ICD-10-PCS** |
| Common/internal carotid or vertebral arteries revascularization, open or endovascular | 03CH, 03CJ, 03CK, 03CL, 03CP, 03CQ |

1. ICD-10-CM = International Classification of Disease, tenth revision, Clinical Modification.
2. ICD-10-PCS = International Classification of Disease, tenth revision, Procedural Coding System.

**Supplemental Table II** All conditions included in the initial analysis stratified by mortality status

| **Concomitant conditions** | **In-Hospital Mortality** | | |
| --- | --- | --- | --- |
|  | *No, Weighted N (%)* | *Yes, Weighted N (%)* | *p-value* |
| Epilepsy and seizures | 32,225 (44.65) | 1,415 (38.61) | 0.002 |
| Status epilepticus | 5,565 (7.11) | 495 (13.51) | < 0.001 |
| Ischemic stroke | 10,295 (14.27) | 1,105 (30.15) | < 0.001 |
| Non-traumatic subarachnoid hemorrhage | 1,780 (2.47) | 190 (5.18) | < 0.001 |
| Intracerebral hemorrhage (non-traumatic) | 3,520 (4.88) | 445 (12.14) | < 0.001 |
| Cerebral edema (non-traumatic) | 8,860 (12.28) | 980 (26.74) | < 0.001 |
| Muscular/Myotonic dystrophies | * | * | * |
| Guillain-Barre syndrome | 205 (0.28) | * | * |
| Unspecified cardiovascular disease | 2,150 (2.98) | 125 (3.41) | 0.507 |
| Cerebral infarction | 8,215 (11.38) | 1,035 (28.24) | <.0001 |
| Occlusion/stenosis of precerebral/cerebral arteries without infarction | 2,745 (3.80) | 120 (3.27) | 0.457 |
| Meningitis, infectious | 175 (0.24) | * | * |
| Encephalitis/myelitis/encephalomyelitis, inflammatory/infectious | 1,020 (1.41) | 150 (4.09) | <.0001 |
| Intracranial/intraspinal abscess/granulomas/phlebitis/thrombophlebitis | 270 (0.37) | * | * |
| Neurodegenerative conditions | 3,325 (4.61) | 210 (5.73) | 0.181 |
| Neuroleptic malignant syndrome | 90 (0.12) | * | * |
| Demyelinating conditions | 865 (1.20) | 60 (1.64) | 0.284 |
| Peripheral neuropathies | 2,865 (3.97) | 105 (2.86) | 0.135 |
| Migraine | 4,295 (5.95) | * | * |
| Cerebral palsy | 90 (0.12) | * | * |
| Autonomic disorders | 535 (0.74) | * | * |
| Coma | 4,435 (6.15) | 570 (15.15) | <.0001 |
| Coma upon arrival to emergency room | 295 (0.41) | 60 (1.64) | <.0001 |
| Coma upon admission | 245 (0.34) | 60 (1.64) | <.0001 |
| Coma 24 hours or more after admission | 105 (0.15) | 75 (2.05) | <.0001 |
| Myasthenia gravis and other neuromuscular junction disorders | 95 (0.13) | * | * |
| Common/internal carotid or vertebral arteries revascularization | 70 (0.10) | * | * |
| Myocardial infarction | 7,230 (10.02) | 470 (12.82) | 0.013 |
| Congestive heart failure | 13,785 (19.10) | 1,005 (27.42) | <.0001 |
| Peripheral vascular disease | 5,045 (6.99) | 295 (8.05) | 0.266 |
| Stroke | 13,274 (91.97) | 3,260 (88.95) | 0.004 |
| Dementia | 6,285 (8.71) | 345 (9.41) | 0.519 |
| Chronic obstructive pulmonary disease | 16,315 (22.61) | 850 (23.19) | 0.714 |
| Rheumatic disease | 4,440 (6.15) | 170 (4.64) | 0.091 |
| Peptic ulcers | 995 (1.38) | 90 (2.46) | 0.017 |
| Liver disease, mild | 3,455 (4.79) | 130 (3.55) | 0.119 |
| Diabetes without complications | 8,915 (12.35) | 500 (13.64) | 0.305 |
| Renal disease, mild | 15,185 (21.04) | 790 (21.56) | 0.738 |
| Diabetes with complications | 12,210 (16.92) | 635 (17.33) | 0.770 |
| Plegia/Paralysis | 5,795 (8.03) | 405 (11.05) | 0.004 |
| Malignancy | 3,735 (5.18) | 405 (11.05) | <.0001 |
| Liver disease, severe | 815 (1.13) | 160 (4.37) | <.0001 |
| Renal disease, severe | 11,060 (15.33) | 630 (17.19) | 0.172 |
| Metastatic tumor | 3,110 (4.31) | 425 (11.60) | <.0001 |
| Human immunodeficiency virus or acquired immune deficiency syndrome | 575 (0.80) | * | * |
| Human immunodeficiency virus | 245 (0.34) | * | * |
| Acquired immune deficiency syndrome | 330 (0.46) | * | * |
| Acute renal failure | 24,270 (33.63) | 2,135 (58.25) | <0.0001 |
| Kidney disorders | 38,145 (52.86) | 2,710 (73.94) | <.0001 |
| Sepsis | 8,745 (12.12) | 1,595 (43.52) | <.0001 |
| Systemic inflammatory response syndrome | 0 (0) | 0 (0) | NA |
| Hypertensive crisis | 29,175 (40.43) | 1,020 (27.83) | <.0001 |
| Reversible cerebral vasoconstriction syndrome | 1,255 (1.74) | 80 (2.18) | 0.373 |
| Hypertensive encephalopathy | 8,720 (12.08) | 280 (7.64) | 0.001 |
| Respiratory failure | 20,590 (28.53) | 2,830 (77.22) | <.0001 |
| Coronavirus disease 2019 | 1,670 (2.31) | 220 (6.00) | <.0001 |
| Toxic encephalopathy | 5,250 (7.28) | 430 (11.73) | <.0001 |
| Primary hypertension | 28,560 (39.58) | 925 (25.24) | <.0001 |
| Resistant hypertension | 0 (0) | 0 (0) | NA |
| Secondary hypertension | 760 (1.05) | * | * |
| Hypertensive heart and chronic kidney disease | 7,465 (10.34) | 530 (14.46) | < 0.001 |
| Aspiration pneumonitis | 5,460 (7.57) | 670 (18.28) | <.0001 |
| Urinary tract infection | 8,700 (12.06) | 535 (14.60) | 0.042 |
| Alcohol related disorders | 5,135 (7.12) | 210 (5.73) | 0.161 |
| Connective tissue diseases with systemic involvement | 3,795 (5.26) | 235 (6.41) | 0.171 |
| Systemic lupus erythematosus | 2,350 (3.26) | 85 (2.32) | 0.163 |
| Malignant neoplasms, including secondary | 3,125 (4.33) | 430 (11.73) | <.0001 |
| Hematological disorders | 34,490 (47.79) | 2,210 (60.30) | <.0001 |
| Acute pancreatitis | 980 (1.36) | 100 (2.73) | 0.003 |
| Body mass index 30 or above | 8,450 (11.71) | 430 (11.73) | 0.985 |
| Transplanted organ and tissue status | 2,090 (2.90) | 140 (3.82) | 0.156 |
| Complications of transplanted organs and tissue | 1,785 (2.47) | 200 (5.46) | <.0001 |
| History of immune checkpoint medications | 0 (0) | 0 (0) | NA |
| Obstetric | 3,670 (5.09) | * | * |

1. NA = not applicable.
2. An asterisk (*) signified unweighted counts of less than 10 and could not be reported per the National Inpatient Sample Data Use Agreement.

**Supplemental Table III** Unadjusted odds ratios for all the clinical variables initially included in the analysis.

| **Clinical Variable** | **Odds Ratio (95% CI)** | **p-value** |
| --- | --- | --- |
| Epilepsy and seizures | 0.78 (0.67, 0.91) | 0.002 |
| Status epilepticus | 1.87 (1.49, 2.34) | <.0001 |
| Ischemic stroke | 2.59 (2.20, 3.06) | <.0001 |
| Non-traumatic subarachnoid hemorrhage | 2.16 (1.54, 3.03) | <.0001 |
| Intracerebral hemorrhage (non-traumatic) | 2.70 (2.13, 3.41) | <.0001 |
| Cerebral edema (non-traumatic) | 2.61 (2.19, 3.1) | <.0001 |
| Unspecified cardiovascular disease | 1.15 (0.76, 1.74) | 0.507 |
| Cerebral infarction | 3.06 (2.58, 3.63) | <.0001 |
| Occlusion/stenosis of precerebral/cerebral arteries without infarction | 0.86 (0.57, 1.29) | 0.457 |
| Encephalitis/myelitis/encephalomyelitis, inflammatory/infectious | 2.98 (2.02, 4.38) | <.0001 |
| Neurodegenerative conditions | 1.26 (0.90, 1.76) | 0.181 |
| Demyelinating conditions | 1.37 (0.77, 2.45) | 0.284 |
| Peripheral neuropathies | 0.71 (0.46, 1.11) | 0.135 |
| Coma | 2.81 (2.28, 3.47) | <.0001 |
| Coma upon arrival to emergency room | 4.06 (2.19, 7.52) | <.0001 |
| Coma upon admission | 4.89 (2.61, 9.16) | <.0001 |
| Coma 24 hours or more after admission | 14.34 (7.39, 27.83) | <.0001 |
| Myocardial infarction | 1.32 (1.06, 1.65) | 0.013 |
| Congestive heart failure | 1.6 (1.35, 1.89) | <.0001 |
| Peripheral vascular disease | 1.16 (0.89, 1.52) | 0.266 |
| Stroke | 0.70 (0.55, 0.89) | 0.004 |
| Dementia | 1.09 (0.84, 1.41) | 0.519 |
| Chronic obstructive pulmonary disease | 1.03 (0.86, 1.24) | 0.714 |
| Rheumatic disease | 0.74 (0.53, 1.05) | 0.091 |
| Peptic ulcers | 1.80 (1.11, 2.92) | 0.017 |
| Liver disease, mild | 0.73 (0.49, 1.08) | 0.119 |
| Diabetes without complications | 1.12 (0.90, 1.39) | 0.305 |
| Renal disease, mild | 1.03 (0.86, 1.24) | 0.738 |
| Diabetes with complications | 1.03 (0.85, 1.25) | 0.770 |
| Plegia/paralysis | 1.42 (1.12, 1.81) | 0.004 |
| Malignancy | 2.28 (1.75, 2.96) | <.0001 |
| Liver disease, severe | 4.00 (2.72, 5.86) | <.0001 |
| Renal disease, severe | 1.15 (0.94, 1.40) | 0.172 |
| Metastatic tumor | 2.91 (2.3, 3.69) | <.0001 |
| Acute renal failure | 2.75 (2.37, 3.2) | <.0001 |
| Kidney Disorders | 2.53 (2.14, 3.00) | <.0001 |
| Sepsis | 5.59 (4.78, 6.53) | <.0001 |
| Hypertensive crisis | 0.57 (0.48, 0.67) | <.0001 |
| Reversible cerebral vasoconstriction syndrome | 1.26 (0.76, 2.1) | 0.373 |
| Hypertensive encephalopathy | 0.60 (0.45, 0.80) | 0.001 |
| Respiratory failure | 8.49 (7.1, 10.15) | <.0001 |
| Coronavirus disease 2019 | 2.70 (1.96, 3.71) | <.0001 |
| Toxic encephalopathy | 1.69 (1.34, 2.14) | <.0001 |
| Primary hypertension | 0.52 (0.43, 0.61) | <.0001 |
| Hypertensive heart and chronic kidney disease | 1.47 (1.19, 1.81) | < 0.001 |
| Aspiration pneumonitis | 2.73 (2.25, 3.32) | <.0001 |
| Urinary tract infection | 1.25 (1.01, 1.54) | 0.042 |
| Alcohol related disorders | 0.79 (0.57, 1.1) | 0.161 |
| Connective tissue diseases with systemic involvement | 1.23 (0.91, 1.67) | 0.171 |
| Systemic lupus erythematosus | 0.71 (0.43, 1.15) | 0.163 |
| Malignant neoplasms, including secondary | 2.94 (2.32, 3.71) | <.0001 |
| Hematological disorders | 1.66 (1.43, 1.93) | <.0001 |
| Acute pancreatitis | 2.04 (1.28, 3.25) | 0.003 |
| Body mass index 30 or above | 1.00 (0.79, 1.26) | 0.985 |
| Transplanted organ and tissue status | 1.33 (0.9, 1.98) | 0.156 |
| Complications of transplanted organs and tissue | 2.28 (1.58, 3.28) | <.0001 |

1. 95% CI = 95% confidence interval
2. OR = Odds ratio
